# Protective Effects of Dietary Grape on UVB-Mediated Cutaneous Damages and Skin Tumorigenesis in SKH-1 Mice

**DOI:** 10.3390/cancers12071751

**Published:** 2020-07-01

**Authors:** Charlotte A. Mintie, Anna K. Musarra, Chandra K. Singh, Mary A. Ndiaye, Ruth Sullivan, Jens C. Eickhoff, Nihal Ahmad

**Affiliations:** 1 Department of Dermatology, University of Wisconsin, 1300 University Avenue, Madison, WI 53706, USA; mintie@wisc.edu (C.A.M.); musarra@wisc.edu (A.K.M.); cksingh@wisc.edu (C.K.S.); mndiaye@dermatology.wisc.edu (M.A.N.); 2 Department of Comparative Biosciences, University of Wisconsin, Madison, WI 53706, USA; rsulliv2@wisc.edu; 3 Department of Biostatistics & Medical Informatics, University of Wisconsin, Madison, WI 53705, USA; eickhoff@biostat.wisc.edu; 4 William S. Middleton VA Medical Center, Madison, WI 53705, USA

**Keywords:** non-melanoma skin cancer, grape, SKH-1, UVB, chemoprevention

## Abstract

Non-melanoma skin cancers (NMSCs) are the most diagnosed cancers in the US and occur more frequently in males. We previously demonstrated chemoprotective effects of dietary grape powder (GP) against UVB-mediated skin tumorigenesis in female SKH-1 mice. To expand on this, here, we determined the effects of GP in a short-term UVB exposure protocol (0 or 5% GP, followed by UVB every other day for 2 weeks) in male and female SKH-1 mice, as well as explored any sex-related differences in UVB carcinogenesis via male SKH-1 mice (0, 3, or 5% GP; UVB twice weekly for 28 weeks). In the short-term study, we found that GP protects against early-stage epithelial hyperplasia and mast cell infiltration in both sexes. In the long term, GP markedly reduced tumor counts and malignant conversion, along with significant decreases in mast cell infiltration, serum IgE and Eotaxin. We also found inhibition of P38 phosphorylation and reduced PCNA, Ki67 and BCL2 levels, suggesting that the anti-inflammatory effects of GP inhibits P38, acting as an upstream regulator to inhibit proliferation and reduce tumor cell survival. Together, GP appears to protect against UVB-mediated skin damage and carcinogenesis in SKH-1 mice and should be explored further as a supplement for NMSC prevention.

## 1. Introduction

Non-melanoma skin cancers (NMSCs) are the most frequently diagnosed cancer, affecting more than 3.5 million Americans annually [1]. The most prevalent NMSCs are basal cell carcinoma (BCC) and squamous cell carcinoma (SCC). Although BCCs and SCCs have lifetime development risks 18–20-fold higher than melanoma, they have low mortality rates (0.12/100,000 for BCC and 0.3/100,000 for SCC) [2]. Current treatments, including resections and chemotherapies, often do not give complete cure, as the recurrence rate following surgical excision is as high as 4.2% after 6.6 years [3]. Interestingly, epidemiological studies have shown a significantly higher incidence of NMSC in men than in women, potentially due to sex-related differences in occupations, lifestyle, and skin barrier functions [4,5]. NMSCs are also associated with significant economic burden, as in the US alone, the national annual expenditures for NMSCs, including both direct and indirect medical costs such as decreases in productivity and loss of potential life-years, are estimated at $4.8 billion [6], underlining the need for more effective treatment and/or prevention options.

Ultraviolet radiation (UVR), particularly its UVB component (290–320 nm), is a complete carcinogen, capable of cancer initiation, promotion and progression, without other agents. UVR is thought to be the primary cause of NMSC development in 90% of cases. Solar UVR penetrates the atmosphere and affects DNA indirectly by stimulating the production of reactive oxygen species (ROS) [7], leading to imbalanced redox homeostasis, inflammation and edema, as well as modulations of biochemical markers and signal transduction pathways [8] such as the Nox and other inflammatory pathways [9]. Direct UVB damage is characterized by formation of cyclobutane pyrimidine dimers (CPDs) and (6,4) photoproducts, which can then overwhelm the nucleotide-excision repair mechanisms and lead to sequence transformations of C→T and CC→TT [10]. Several key cancer-related pathways have been shown to be affected by UVB, including TP53 [11,12], epidermal growth factor (EGF) [13], mitogen-activated protein kinases (MAPKs) [14], and phosphatidylinositol 3-kinase (PI3K) [13,15]. Additionally, DNA damages caused by UVR can deregulate cytokine levels, including interleukin-1 (IL-1) and tumor necrosis factor-alpha (TNF-α), leading to chronic inflammation [16]. This may ultimately lead to the development and progression of cancer via apoptotic resistance and enhanced cell proliferation and survival [16]. In fact, inflammation has been recognized as a hallmark of cancer and nuclear factor-κB (NF-κB) signaling as a key player linking chronic inflammation to cancer [17].

Currently, the major means to prevent DNA damage from UVR are the proper use of sunscreen and limiting sun exposure [18]. However, the rising incidence of skin cancer diagnoses suggests a need for additional strategies for NMSC management. One such approach is chemoprevention with dietary antioxidants, such as grape antioxidant resveratrol, which has been extensively studied for its health and benefits and chemopreventive effects [19,20]. It has been shown that the antioxidants found in grapes, including stilbenes (resveratrol), flavonols (quercetin, kaempferol, isorhamnetin, taxifolin) catechins (catechin, epicatechin), and anthocyanins (cyanidin, peonidin, malvidin), may prevent or mitigate many health conditions including cancer [21,22]. The multitude of health benefits related to the biochemistry of grapes suggests that grapes could be useful in preventing skin cancer, especially when used in its whole food form due to the possibility of synergistic activity between the several antioxidant ingredients and other bioactive compounds they contain [21,22,23]. Recently, we demonstrated the chemoprotective effects of dietary grape powder (GP) in a long-term UVB-mediated skin tumorigenesis setting in female SKH-1 mice, where we showed that GP supplementation increased DNA damage repair, decreased cell proliferation, and oxidative stress, and increased apoptotic response and ROS metabolism [24]. However, additional studies in different models and conditions are necessary to validate these findings before embarking on human studies. In this study, employing a short-term UVB protocol, we determined the effects of dietary GP on male as well as female SKH-1 hairless mice. In addition, employing a long-term UVB protocol of skin carcinogenesis, we also determined the effect of GP feeding on NMSC formation in male SKH-1 hairless mice to determine any possible biological variability in the observed responses due to sex.

## 2. Results and Discussion

### 2.1. Dietary GP Consumption Decreases Epidermal Thickening and Mast Cell Counts in SKH-1 Hairless Mouse Skin in a Short-Term UVB Exposure Protocol

**Figure 1:**
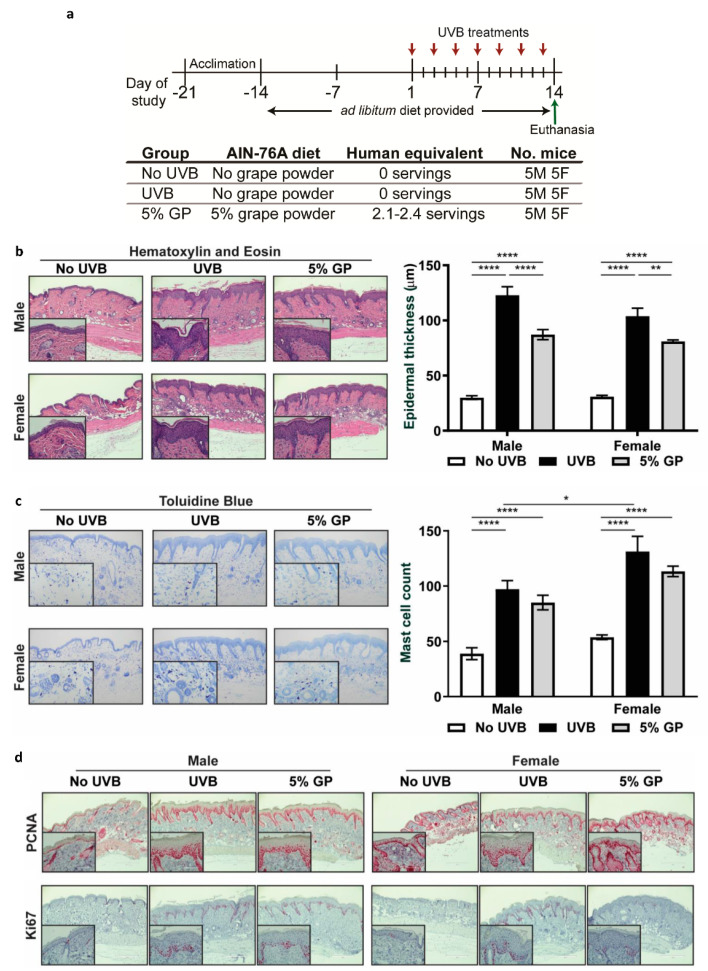
Dietary GP consumption results in UVB-mediated cutaneous hyperplasia and mast cell infiltrations in SKH-1 hairless mice. (**a**) Timeline of short-term UVB-mediated cutaneous damage study. (**b**) GP consumption significantly reduced epidermal thickness as determined by measuring epidermal thickness from the base of the stratum basale to the base of the stratum corneum on H&E-stained tissue sections. (**c**) Mast cell infiltration shown by toluidine blue staining. At least five images were taken at 10x magnification across five different skin sections of each mouse. Mast cells (violet metachromatic cytoplasmic granules) were counted using ImageJ, and then averaged per mouse. (**d**) Expression of proliferative markers PCNA and Ki67 was assessed using immunohistochemical staining techniques. Representative sections are shown. All images are represented at 10× magnification, with inset at 40× magnification. The data represent the mean ± SEM of all five animals per group. A two-way ANOVA with Tukey’s multiple comparison test was performed (* *p* <0.05, ** *p* <0.01, **** *p* < 0.0001).

UVB skin exposure leads to a cascade of events that may result in skin damage and carcinogenesis. Here, we sought to evaluate the benefits of GP consumption using a short-term UVB exposure protocol (total six exposures of 180 mJ/cm^2^, in two weeks) to represent an outdoor sunny vacation. An equal number of male and female mice were divided into three groups: (1) no UVB: control diet without UVB; (2) UVB: control diet with UVB; (3) 5% GP: 5% GP diet with UVB. After two weeks of GP consumption, 180 mJ/cm^2^ UVB was given every other day for 2 weeks (Figure 1a). As previously observed [24], GP supplementation was well tolerated in all mice. The average daily GP consumption in the 5% GP group corresponded to 239.8 ± 11.3 and 197.5 ± 4.1 mg for males and females, respectively. These correspond to human equivalent doses of 58.3 and 48.0 g/day, based on a dose translation model [25], or 2.4 and 2.07 servings of grapes.

Studies have shown that following UVB exposure, an upregulation of tumor suppressor TP53 causes a G1 phase cell cycle arrest, allowing keratinocytes to recover from DNA damage or undergo apoptosis [26,27]. In addition, cells enter a hyperproliferative state to thicken the epidermis, thereby protecting it against further UVB-mediated damages. Therefore, we determined the effect of GP consumption on UVB-mediated epidermal thickening using H&E-stained dorsal skin. Both in male and female mice, we found that UVB exposure resulted in a significantly thickened epidermis compared to the control mice (Figure 1b). Further, 5% GP feeding resulted in significant decreases in UVB-mediated increases in epidermal thickening in mice (Figure 1b). Following the onset of hyperplasia, UVB can induce an inflammatory response [27]—more specifically, the infiltration of inflammatory mast cells. Mast cells release soluble mediators (i.e., IL-10, TNF-α and VEGF) and are known to participate in tissue remodeling, repair, and wound healing [28,29]. We observed significant increases in mast cell infiltration in all UVB-treated groups, with trending reductions in the 5% GP groups from UVB control mice (Figure 1c). Reduced mast cell infiltration has also been observed after topical application of grape stem extract in UVB-exposed C57BL mice [30]. Interestingly, mast cell infiltration in the UVB group was significantly higher in females than males, although not in the 5% GP group. This supports previous findings that female SKH-1 mice exhibit higher levels of inflammation [31]. Next, the effects of treatments were assessed on proliferative index by measuring the levels of proliferative markers PCNA and Ki67 in skin by immunohistochemical staining. Upon visual examination, the qualitative pattern of expression in both groups treated with UVB appeared to be expanded in basal regions of skin, consistent with the enhanced proliferation and epidermal thickening observed in UVB-treated skin. However, treatment with GP did not result in overt changes in the histologic pattern of PCNA and Ki67 expression (Figure 1d). Although significant changes were not observable, the distinct trend towards reduced epidermal thickening and mast cell infiltration may provide insight into the short-term benefits of GP consumption in protecting against UVB damages.

### 2.2. Dietary GP Consumption Reduced Chronic UVB Exposure-Mediated Skin Tumorigenesis in Male SKH-1 Hairless Mice

**Figure 2:**
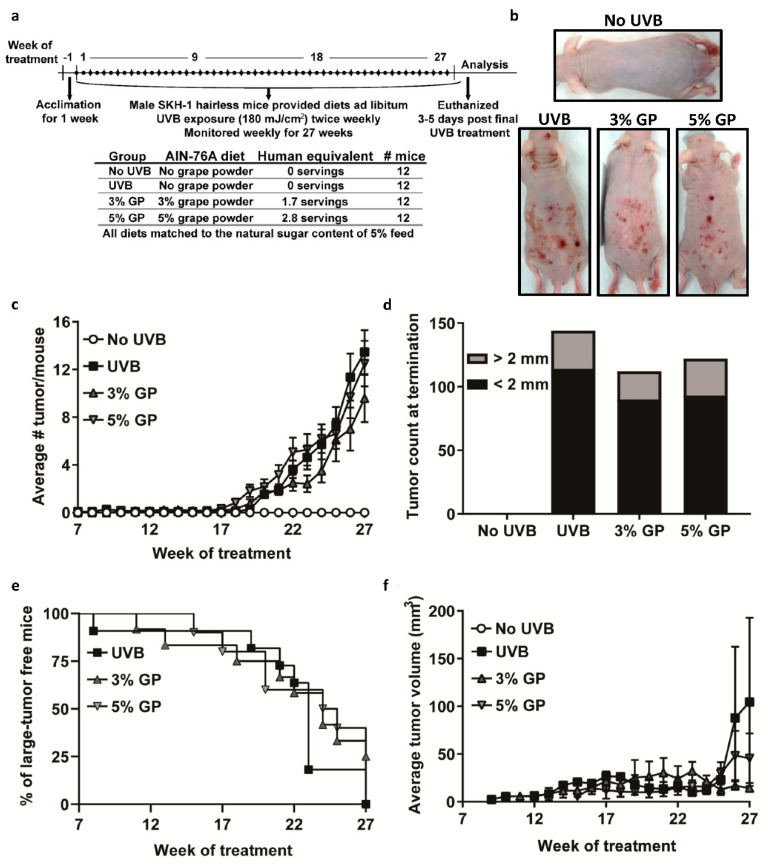
Dietary GP consumption reduces UVB-mediated skin carcinogenesis in male SKH-1 hairless mice. (**a**) Timeline of chronic UVB-mediated carcinogenesis study. (**b**) Representative SKH-1 mice from each group at the end of the study. (**c**) Average number of total tumors per mouse per group. (**d**) Additive large (>2 mm diameter, grey) and small (<2 mm diameter, black) tumor count at termination of the experiment. (**e**) Large tumor latency shown as the percentage of mice without measurable tumors (>2 mm in diameter). (**f**) Average tumor volume of measurable tumor per group. All data are presented as the mean ± SEM.

Although no registries document the current incidence of NMSC cases in the US, epidemiological studies have reported that cases of NMSC are significantly higher in men than women [4,5]. Previously, we demonstrated the long term effects of GP consumption against UVB-mediated carcinogenesis in female SKH-1 hairless mice [24]. Here, we sought to determine the effects of GP on UVB skin carcinogenesis in male SKH-1 mice utilizing the same initiation-promotion protocol (180 mJ/cm^2^ UVB twice weekly for 28 weeks) (Figure 2a), since it is known that males tend to produce larger skin tumors that may appear earlier compared to females, upon UVB exposure [31]. In this study, we provided the same GP diet ad libitum to the mice as given in our previous female mouse study [24]. As expected, the males consumed more food per day than females did in our previous study, resulting in a higher dose of GP per day (160 ± 2.7 and 268 ± 6.3 mg for 3% and 5% GP groups, respectively). This would be equivalent to 1.70 or 2.84 servings of grapes per day in humans, versus the 1.07 and 1.85 servings of grapes in the female mice [24].

Over the course of the study, all mice treated with UVB developed tumors (Figure 2b). The first small discernable papules (small tumor <2 mm in diameter) arose at weeks 7, 10, and 14 for the 0%, 3%, and 5% GP groups, respectively. Our statistical model of the observed reduction in average tumor burden per mouse, including papules and large tumors, was not significant at 27 weeks, as the average tumor count per mouse was 13.46 ± 1.85 for UVB, 9.58 ± 1.99 for 3% GP, and 12.50 ± 1.9 for 5% GP (Figure 2c). When taking into account the total tumor count at termination, we observed marked reductions of 22% (for 3% GP) and 16% (for 5% GP), when compared to UVB-exposed control counterparts (Figure 2d). When papule growth exceeded 2 mm in diameter, a digital caliper was used to measure and track the weekly growth of the tumors. The first measurable tumors were observed at weeks 8, 11, and 15 for the 0, 3% GP, and 5% GP groups, respectively; and by week 24, the large tumor incidence rates were 82%, 50%, and 40% (Figure 2e). By week 27, the average tumor volume was significantly decreased in both GP groups (3% GP, 14.26 ± 3.74 mm^3^, *p*-value = 3.31 × 10^−12^; 5% GP, 45.51 ± 26.1, *p*-value =1.74 × 10^−5^) versus UVB (104.52 ± 88.2) (Figure 2f). Utilizing a mixed model of linear regression, we also observed that 3% GP group tumor volume was significantly lower than the 5% GP group at 27 weeks. Interestingly, by the end of the study three mice in the 3% GP group did not have measurable tumors. As we observed many unique and varied tumors, we sought to determine whether GP reduces the capacity of the tumor to convert into a malignant phenotype.

### 2.3. Dietary GP Consumption Reduces the Malignant Conversion of UVB-Mediated Tumors in SKH-1 Hairless Mice

**Figure 3:**
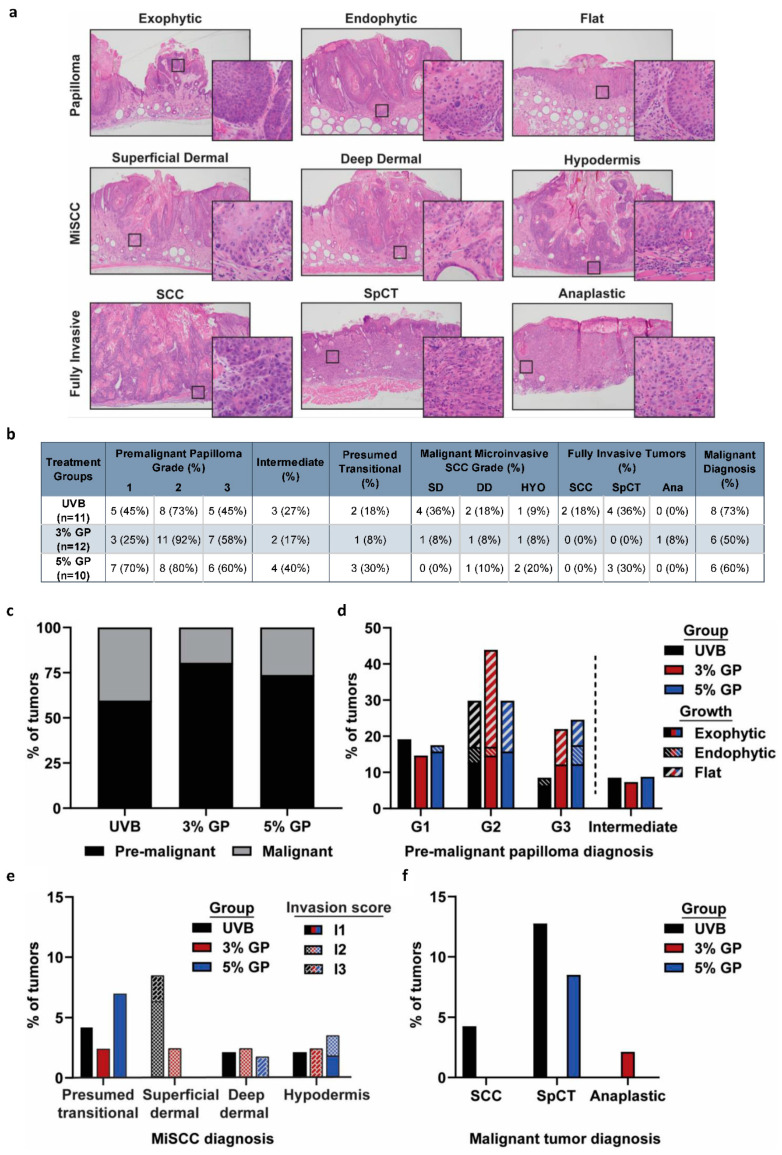
Dietary GP consumption reduces malignant conversion of tumors in UVB-treated SKH-1 hairless mice. (**a**) Images of exemplar lesions of each diagnostic category of tumors. (**b**) Percentages of mice within each treatment group exhibiting lesions in each of the histologic-based classes. (**c**) Tumor diagnoses fell into two categories of premalignant (black) or malignant (grey) tumors. (**d**) Premalignant tumors were papillomas diagnosed based on grade and growth patterns. Grading was assigned based on the pattern of growth and degree of atypia (G1 to G3, minimal-moderate-marked). Papillary growth patterns relative to the dermis were as follows, outward (exophytic), flat, and inward (endophytic). Premalignant intermediate lesions were also observed. (**e**) Malignant microinvasive SCC (MiSCC) were identified based on the presence of stromal invasion and categorized based on their depth of extension into the dermis and subcutis. Invasion extent was also scored based on the amount of invasive extension into surrounding stroma (I2 to I3, mild-moderate). Presumed transitional lesions exhibited increasingly malignant features that surpassed features of premalignant lesions but failed to unequivocally display the clear invasive growth that defines MiSCC and were included among the malignant lesions for stringency of analysis. (**f**) Fully invasive tumor diagnosis. SCC diagnosed upon invasion into the panniculus carnosus. Spindle cell carcinomas (SpCC) and one anaplastic tumor diagnosed based on their morphology, rather than the depth of invasion. All data columns are represented as the percentage of total tumors diagnosed per group.

To further determine the effects of treatments on disease progression and to classify the lesions, representative lesions of each size were processed for histologic evaluation. The lesions were scored based on criteria previously described by Benavides et al. [32] with additional criteria added as described in the Section 3. Papillomas were determined to be premalignant lesions, whereas microinvasive SCC (MiSCC), fully invasive SCC, spindle cell tumor (SpCT), and anaplastic tumors were deemed malignant. Additionally, we included a class of “Presumed transitional” lesions that exhibited increasingly malignant features that surpassed features of premalignant lesions, but failed to unequivocally display the clear invasive growth that defines MiSCC. For maximum stringency, “presumed transitional” lesions were also categorized as malignant MiSCC because our study is a prevention study, and underreporting malignancy would bias our results towards observing a favorable effect of our intervention. Images percentage of exemplar lesions in each of the diagnostic categories are shown in Figure 3a. Percentages of mice within each treatment group exhibiting lesions in each of the histologic classes are shown in Figure 3b. Of the tumors graded, 81% and 74% of the tumors in 3% and 5% GP diet groups, respectively, were diagnosed as premalignant compared to 60% of tumors in the UVB control group (Figure 3c). The most prevalent premalignant lesions were G2 papillomas across all groups with an incidence rate of 29.7%, 43.9%, and 28.82% for UVB, 3% GP, and 5% GP, respectively (Figure 3d). In each group, premalignant intermediate tumors, which were lesions classified as premalignant in one blinded assessment and as early malignant in a second independent blind evaluation and ultimately classified as premalignant, were observed with similar incidence. Although all mice developed premalignant lesions, we observed a marked reduction in the malignant conversion in both GP treatment groups. In total, only 50% and 60% of mice in 3% and 5% GP groups developed malignant lesions versus 73% of the UVB animals (Figure 3b). The majority of malignant lesions in both GP treatment groups were MiSCC, 93.3% (3% GP) and 84.6% (5% GP), as compared to 73.3% of the UVB counterparts (Figure 3b,e). However, 17% of the tumors diagnosed in the UVB control group were fully invasive SCC or SpCT, as compared to no SCCs present in either treatment group, although one anaplastic tumor was diagnosed in the 3% GP group and SpCT were present in the 5% GP group (7.0%) (Figure 3f). These findings suggest that GP consumption slows tumor progression and malignant conversion when compared to UVB control counterparts, consistent with the findings of reduced tumor volume and burden. The increased tumor volume in the UVB control group is consistent with the trend for malignant tumors.

**Figure 4:**
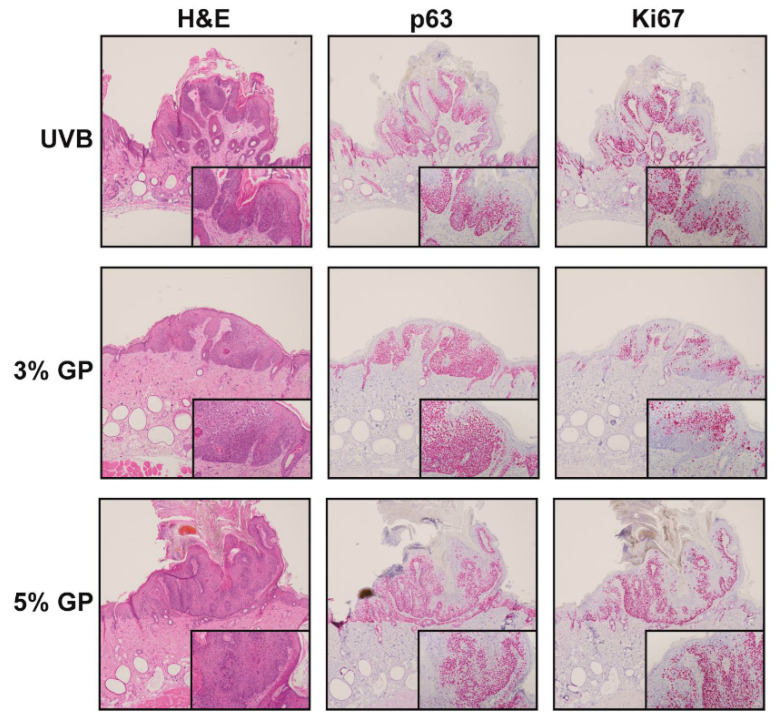
Dietary GP consumption does not affect premalignant growth patterns following long-term UVB exposure in SKH-1 hairless mice. Histological analysis of exophytic papillomas with a G2 atypia score from the UVB-treated groups. Staining includes hematoxylin and eosin (H&E), epithelial marker p63, and proliferative marker Ki67. Images were obtained at 4× magnification and inset at 20× magnification.

To further characterize the lesions, we performed immunostaining for epithelial marker p63 and proliferation marker Ki67. The SKH-1 hairless mouse model is known for developing SCC that resemble human tumors [32], which show enhanced levels of p63 (a transcription factor in the TP53 family) in the basal proliferative layer within the skin [33,34]. Although staining was performed on the majority of tumors, Figure 4 shows staining from representative grade 2 exophytic papillomas diagnosed from hematoxylin & eosin (H&E) staining, selected based on the highest frequency of diagnosis across the majority of groups, except for the 3% GP group, having the highest incidence of grade 2 flat papillomas. Our evaluation of low-grade tumors served to reduce the bias of expression in all groups, as higher-grade tumors are not prevalent in both GP treatment groups. We did not observe differences in patterns of p63 protein expression among the groups. Patterns of Ki67 expression, a marker indicative of cell proliferation, were also similar among the groups, suggesting a need for assessing markers and pathways relevant to tumorigenesis.

### 2.4. Dietary GP Consumption Imparts Anti-Inflammatory Effects in Long-Term UVB-Treated SKH-1 Hairless Mice

**Figure 5:**
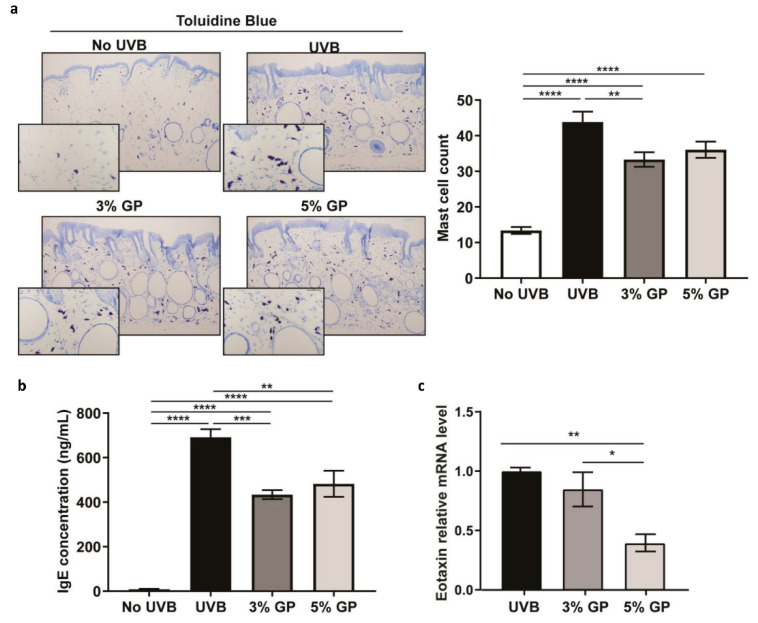
Dietary GP consumption reduces dermal mast cell presence, serum IgE and Eotaxin expression. (**a**) Mast cells were identified by toluidine blue staining in the dermal region of the dorsal skin of the no UVB groups and the involved skin directly adjacent to tumors of all groups treated with UVB. Images are at 10× magnification, with inset at 40× magnification to show the appearance of mast cells. Mast cell number was quantified from five images at 20× magnification per animal using ImageJ program and averaged. The data represent the mean ± SEM of all animals per group. (**b**) Total serum IgE concentration was determined by ELISA. (**c**) RT-qPCR analysis of Eotaxin expression in papules. Data are presented as the mean ± SEM of three biological pools of three animals per group (n = 9) in technical triplicate. A one-way ANOVA with Tukey’s multiple comparison test was performed (* *p* < 0.05, ** *p* < 0.01, *** *p* < 0.001, **** *p* < 0.0001).

Cutaneous inflammation, as well as angiogenesis and metastasis within tumors, have been implicated as key players in promotion and progression of NMSCs [16,35]. Interestingly, several grape chemopreventive agents are known to affect multiple signaling molecules related to tumor angiogenesis and metastasis [36]. Previously, employing tandem mass tagging LC–MS/MS in tumor samples from our female model of UVB-induced carcinogenesis, we demonstrated that GP attenuates the chronic pro-inflammatory environment in skin tumors [37]. However, Thomas-Ahner et al. demonstrated that not only do male SKH-1 mice develop more histologically advanced tumors than females, but the two sexes have different amounts of DNA damage and inflammation [31]. Studies suggest that UV irradiation increases the density of mast cells, which are required for UV-induced immune suppression leading to skin cancer susceptibility [38]. We used toluidine blue to stain mast cells within the dermis of all mice. Toluidine blue may also stain basophils present in the skin, but basophils are generally a low abundance leukocyte in the circulatory system, whereas mast cells mature in tissue [39]. Upon quantifying mast cell number in the UVB-exposed dermis, we observed a marked decrease in mast cell infiltration in both GP treatment groups (Figure 5a). The role of mast cells in skin cancers is somewhat controversial, with no clear evidence of mast cells having clear tumor promoter or tumor suppressor function [29]. However, it is suggested that the cross-taThelk between mast cells and sensory nerve fibers in photo-damaged skin may affect NMSC prevalence (reviewed in [40]). It is possible that the reduced accumulation of mast cells observed in our study may be due to anti-inflammatory effects of GP. As an activator of mast cells, Immunoglobulin E (IgE), known to be upregulated in allergy responses [41], was evaluated based on the observation that all mice treated with UVB appeared to have pruritus, or extensive itching, resulting in scratch and bite wounds throughout the study (data not shown). It is known that elevated human IgE levels may be indicative of increased susceptibility to subsequent SCC lesions [42]. Although we cannot predict the extent to which serum IgE might activate mast cell infiltration, we observed significant decreases in IgE in both GP treatment groups (Figure 5b). This is in accordance to a reported study where the grape antioxidant resveratrol was shown to inhibit IgE-sensitized mast cells and passive cutaneous anaphylaxis in mice [43].

Additionally, we utilized serum to profile cytokines potentially linked to the chemopreventive response using the Cytokine and Chemokine 36-Plex Mouse ProcartaPlex Panel 1A using magnetic beads on a Luminex platform. Although it can be difficult to detect minute changes in cytokine response using this platform, we observed slight decreases in serum Eotaxin (CCL-11) in 5% GP treatment group (data not shown). We also evaluated Eotaxin mRNA expression within small papules using RT-qPCR analysis, and found significant decreases in Eotaxin in GP treatment groups (Figure 5c).

**Figure 6:**
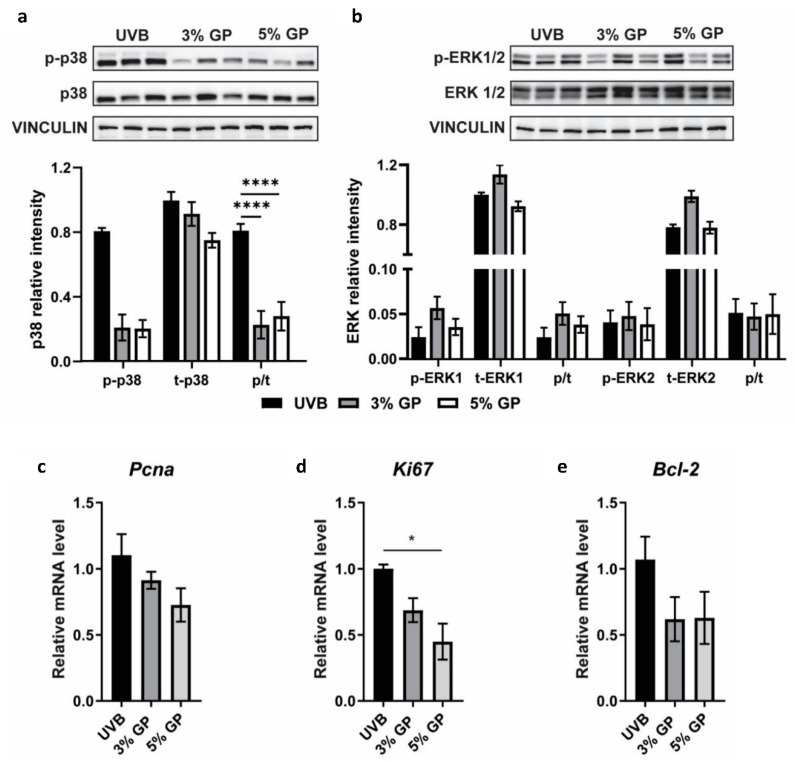
Dietary GP consumption reduces phosphorylation of p38 and reduces proliferation and pro-survival markers. (**a**,**b**) Immunoblot analysis of MAPK proteins (**a**) p38 and (**b**) ERK1/2. Vinculin (VIN) was used as a loading control. Statistical analysis was performed on the p/t ratio, which indicates the ratio of phosphorylated/total protein. (**c**–**e**) RT-qPCR analysis of proliferative markers (**c**) Pcna and (**d**) Ki67, and (**e**) pro-survival marker Bcl-2. Data are represented as the mean ± SEM. A one-way ANOVA with Tukey’s multiple comparison test was performed (* *p* < 0.05, **** *p* < 0.0001) (For Western Blot raw data, please refer to the Supplementary Materials Figure S1).

Next, we sought to determine whether GP acts on inflammatory mediators linked to advancing skin tumorigenesis, such as the MAPK signaling pathway, which has been previously linked to grape antioxidant resveratrol’s biological response and is known to control cellular proliferation, differentiation, and apoptosis [22]. Additionally, in our previous proteomics study evaluating the anti-tumorigenic effect of dietary grape in female SKH-1 tumors, we found that acute phase response (APR) signaling was abated with the consumption of GP. Upon evaluation of upstream regulators of APR in the female mice, including ERK1/2 and NF-κB, we found that anti-inflammatory effects of GP were associated with modulation in these pathways leading to inhibition of tumor growth [37]. Therefore, we performed immunoblot analysis of phosphorylated and total p38 and ERK1/2 to elucidate if GP modulates MAPK pathway during protection from UVB-mediated skin tumorigenesis. Our data show that UVB-mediated phosphorylation of p38, but not ERK1/2, was reduced by GP consumption in low-grade papules (Figure 6a,b). Studies have shown that p38 plays a role in controlling cell proliferation in head and neck SCC and activation is more prominent in less differentiated tumors associated with poor prognosis, whereas the accumulation of activated ERK1/2 was not as prominent [44]. Therefore, we suspect that dietary GP acts upon p38 signaling to reduce cell proliferation and tumor growth. As accumulated p38 expression is linked to increased cellular proliferation, we also assessed the expression of proliferative markers Pcna and Ki67 via RT-qPCR using cDNA from papules. We observed a marked reduction in both markers in GP treatment groups (Figure 6c,d). We also observed a marked reduction in pro-survival Bcl-2 in the papules (Figure 6e). Collectively, we believe that the anti-inflammatory capacity of dietary GP on early-stage, low-grade papules inhibits MAPK signaling, and therefore acts as an upstream regulator to limit proliferation and survival of tumor cells.

## 3. Materials and Methods

### 3.1. Materials and Animal Care

Freeze-dried grape powder (GP) was provided by the California Table Grape Commission, (Fresno, CA, USA). This proprietary mix is composed of fresh red, green, and black grapes cultivated in California. The diet was formulated in the AIN-76A base diet by Envigo (Madison, WI, USA) as described in [24]. The control diet contained no GP, whereas the 3% and 5% GP diets contained the indicated amount of GP (*w*/*v*). All diets were sugar matched to the natural content of the 5% diet. For our calculations, 23 g of GP is equivalent to one ¾ cup serving of whole grapes.

All animal experiments were approved by the University of Wisconsin Institutional Animal Care and Use Committee. SKH-1 Elite mice (Crl:SKH-1-Hrhr, Strain 477) were purchased from Charles River Laboratories (Wilmington, MA, USA) at 5 weeks of age and allowed to acclimate for one week prior to study initiation. Animals had access to water and feed ad libitum. Throughout the experiments, all animals were monitored for general health, body weight, and food consumption. A calibrated Research Irradiator (Daavlin Company, Bryan, OH, USA) was used to administer measured doses of UVB for all studies.

### 3.2. UVB-Mediated Cutaneous Damage

Male and female SKH-1 hairless mice (n = 5 per sex, per group) were separated into the following groups: (1) control diet with no UVB, (2) control diet with UVB, and (3) 5% GP diet with UVB. Diets were provided to mice 2 weeks prior to the first dose of UVB. A dose of 180 mJ/cm^2^ was given every other day for a total of 2 weeks. This UVB exposure protocol may represent a sun-exposure during a sunny vacation (for example on a beach). All mice were euthanized 24 h after the seventh dose of UVB and skin samples were formalin-fixed and submitted for histology. Additional skin samples were snap-frozen in liquid nitrogen, and then stored at −80 °C until further processing.

### 3.3. Histology and Epidermal Thickness

Formalin-fixed tissues were paraffin-embedded, sectioned, and mounted on serial slides at the UW Translational Research Initiatives in Pathology (TRIP) laboratory. One serial slide from each mouse was stained with hematoxylin and eosin (H&E) for histological determination.

Epidermal thickness was assessed by analyzing five fields of view from H&E-stained dorsal skin sections of each mouse using an EVOS XL Core Cell Imaging System (ThermoFisher Scientific, Waltham, MA) and quantified using ImageJ software. Five measurements from the basement membrane to the base of the stratum corneum were obtained from five dorsal views and averaged per animal.

### 3.4. Toluidine Blue Staining

Tissue slides were deparaffinized in xylenes, and then rehydrated in a standard ethanol gradient followed by running water. Slides were then stained for 3 min in fresh toluidine blue (5 mL of 1% toluidine blue O (Millipore Sigma, Burlington, MA, USA) in 70% ethanol, mixed with 45 mL 1% sodium chloride (pH 2.0–2.5)). Slides were washed in distilled water, dehydrated, cleared in xylenes and cover slipped. Mast cells were quantified from five images of dorsal skin sections by counting violet cells at 10–20× magnification. Representative images were obtained using Nikon Plan Apo lenses at 10× and 40× magnification on a Nikon Eclipse Ci microscope equipped with a Nikon DsRi2 digital color camera (Nikon, Minato City, Tokyo, Japan).

### 3.5. UVB-Mediated Carcinogenesis

Male SKH-1 mice (n = 12 per group) were grouped as follows: (1) control diet with no UVB, (2) control diet with UVB, (3) 3% GP diet with UVB, and (4) 5% GP diet with UVB. Diets were provided on the first day of UVB exposure at six weeks of age. Mice in groups 2–4 were irradiated with 180 mJ/cm^2^ UVB twice weekly for 27 weeks. Distinguishable tumors were counted and location noted weekly and at the time of euthanasia. If a tumor disappeared in less than two weeks, it was determined to be a papule and eliminated from the dataset. When tumor diameter exceeded 2 mm, a digital caliper was used to measure the tumor, and volume was calculated by l*w*h*(π/6). Mice were euthanized 3–5 days after their last UVB exposure. Immediately after euthanasia, blood was collected via cardiac puncture and allowed to clot for 30 min before centrifugation at 1500× *g* for 15 min at RT for the collection of serum. Dorsal skin adjacent to the tumors and cross-sections of a subset of both large tumors and papules with intact adjacent skin were formalin fixed and submitted for histology. Other tumors and adjacent skin samples were snap-frozen in liquid nitrogen, and then stored at −80 °C until further processing.

### 3.6. Lesion Scoring

Tumors were collected with flanking dorsal skin to ensure lesions were well oriented in cross-section and through full-thickness skin. One H&E-stained slide of skin samples from each mouse in the UVB-mediated tumorigenesis study was examined and neoplastic lesions were scored in a blinded manner by a board-certified veterinary pathologist (R.S.). Some groups of lesions were scored independently twice to verify the diagnosis. Grading guidelines were similar to those outlined in [31,32] with the following additions. Exophytic papillomas encompassed flat epithelial lesions lacking a pronounced papillary pattern and lacking atypical cells (grade 1) through exophytic papillary masses with some atypical cells (grade 3) as described in Benavides et al. [32] We also observed hyperplastic epidermal lesions with dysplasia and cellular atypia that tended towards sessile (flat) or inward (endophytic) growth. We scored these as flat and endophytic papillomas, respectively, with increasing dysplasia and atypia reflected as grades 2 and 3. Although the flat and some endophytic lesions could have histologic features that could resemble a lesion class termed “pseudoepitheliomatous hyperplasia” by Voigt et al. and described as associated with cutaneous ulceration [45], in the absence of histologic evidence of association of the lesions observed in our study with skin ulcers, we utilized the designation “papilloma” for clarity. The exophytic, endophytic, and flat papilloma lesions were considered premalignant. An additional group of lesions that were scored as high-grade preneoplastic lesions in one blinded review of the slides and as “presumed transitional” lesions on a second blinded review were categorized as “intermediate” and were included among the premalignant lesions. The lesions categorized as “presumed transitional” exhibited increasingly malignant features that surpassed features of premalignant lesions and/or angular extension (invasion) into the surrounding connective tissue (invasion) that was either minimal or equivocal. These were interpreted to be lesions transitioning from the premalignant group into the microinvasive squamous cell carcinoma (MiSCC) class. We observed MiSCC as described in Benavides et al. [32] and characterized these both by their depth of penetration into the superficial dermis (SD, ~50% of dermis superficial to dermal cysts), deep dermis (DD, ~50% of dermis deep to the level of dermal cysts), or hypodermis (HYO), and also by their extent of invasion into surrounding connective tissue (degree 2 = mild, degree 3 = moderate). In instances when the hypodermis was very thin or not evident, tumors that extended beneath the deep dermis were classified as HYO. As described by previous authors [32], we observed SCCs, spindle cell tumors (SpCT), and one anaplastic tumor. SpCT tumors were vimentin positive (data not shown). Tumors were only scored as SCC if they invaded into the panniculus carnosus muscle as described while SpCT and anaplastic tumors were scored based on morphology regardless of depth of penetration. miSCC, SCC, SpCT, and anaplastic lesions were categorized as malignant lesions.

### 3.7. Immunohistochemistry (IHC)

Slides were deparaffinized in xylenes and rehydrated in a standard ethanol gradient. Slides were steamed in 1× IHC Epitope retrieval solution (IHC World LLC, Woodstock, MD, USA), followed by washing with tris-buffered saline with 0.1% Tween-20 (TBS-T). Slides were blocked using 5% normal goat serum followed by incubation in primary antibody (PCNA (Invitrogen, Carlsbad, CA, USA; #PA5-27214, 1:500), Ki67 (Cell Signaling, Danvers, MA, USA; #12202, 1:500) and p63 (Proteintech, Rosemont, IL, USA; #12143, 1:250)) in TBS-T overnight at 4 °C in a humidified chamber. Next day, slides were washed, incubated in secondary antibody and ABC-AP per the manufacturer’s instructions using Vectastain ABC Kit (Vector Labs, Burlingame, CA, USA). Slides were then exposed to Vector Red Alkaline Phosphatase Substrate Kit (Vector Labs) until desired staining intensity was observed (7–15 min) and counterstained using Hematoxylin QS (Vector Labs) for 8 s. The slides were rinsed in running tap water, dehydrated via standard ethanol series, cleared by two 5 min rounds of xylenes, and coverslipped for imaging in xylene:permount (1:1).

### 3.8. Protein and RNA Isolation

Tumor tissues (small papules) were ground to a powder in liquid nitrogen before being divided for protein and RNA isolation. For protein isolation, the powdered tissue was lysed in RIPA buffer (Millipore) with freshly added PMSF (Amresco/VWR, Radnor, PA, USA), and both phosphatase and protease inhibitor cocktails (ThermoFisher Scientific, Waltham, MA, USA). Samples were then placed on ice and homogenized using a Kinematica Polytron PT 2500 E (ThermoFisher Scientific) for 5 s intervals until fully disrupted. Tissues were lysed with intermittent vortexing prior to centrifugation at 15,000× *g* for 30 min at 4 °C. Supernatants were collected and protein concentration was determined by BCA Protein Assay (ThermoFisher Scientific) per the manufacturer’s protocol. For RNA isolation, the RNeasy Fibrous Tissue Mini Kit (Qiagen, Hilden, Germany) was used per the manufacturer’s protocol.

### 3.9. RT-qPCR Analysis

RNA from three mice were pooled per sample in three separate groupings to represent the biological averages across the cohort. RNA was transcribed using random primers and M-MLV reverse transcriptase (Promega). RT-qPCR was then performed with SYBR Premix Ex Taq II (TaKaRa, Mountain View, CA, USA) and appropriate primer sets (Eotaxin, Pcna, Ki67, Bcl-2 and Gapdh) retrieved from Primer Bank [46]. Relative target mRNA levels were calculated using ΔΔCT comparative method and Gapdh as an endogenous control.

### 3.10. IgE ELISA

IgE levels were examined using mouse IgE ELISA (Abcam, Cambridge, UK) per the manufacturer’s instructions. Three biological replicates per group, serum pooled from three mice each, were examined in technical triplicate. Serum pools were diluted 1–50 in the diluent buffer provided prior to application on the plate. The plate was read at λ = 450 nm on a BioTek Synergy H1 (Winooski, VT, USA) plate reader.

### 3.11. Cytokine Array

The expression analysis for various cytokines was performed using the Invitrogen Cytokine and Chemokine 36-Plex Mouse ProcartaPlex Panel 1A (ThermoFisher Scientific) per the manufacturer’s instructions. Three biological replicates per group, serum pooled from three mice each, were examined in technical duplicate. The plate was analyzed using a Luminex MagPix (Luminex Corporation, Austin, TX, USA). The raw data, with bead lot information, were then imported into the ProcartaPlex Analyst Software (ThermoFisher Scientific) for data analysis.

### 3.12. Immunoblot Analysis

Immunoblotting was performed as described previously [24]. Primary antibodies from Cell Signaling were used (p-p38 #4511, p38 #9212, pERK1/2 #9101, ERK1/2 #9102, and Vinculin #4650; all 1:1000). Densitometry was performed to determine band intensity using Adobe Photoshop CC 2015. Background intensity was subtracted from the band intensity prior to normalization (target/loading control). Lanes were normalized against the average intensity of control lanes (lane/(average of lanes1,2,3)).

### 3.13. Statistical Analysis

All outcome parameters were summarized in terms of the mean ± standard error of the mean (SEM). Analysis of variance (ANOVA) was conducted to compare mast cell counts, IgE levels, relative mRNA expression and immunoblot analysis parameters between treatment groups. Values for each group were analyzed in GraphPad Prism Software (San Diego, CA, USA) using a one-way ANOVA with Tukey’s multiple comparisons test for conducting pairwise comparison between groups. Average numbers of tumors were analyzed using a negative binomial regression model with animal-specific random effects. A linear mixed-effects model with animal-specific random effects was used to analyze changes in tumor volume over time. Model assumptions were verified by examining residual plots. Kaplan-Meier analysis was conducted to analyze tumor-free survival. Comparisons of tumor-free survival between groups were performed using the log-rank test. All reported *p*-values are two sided and *p* < 0.05 was used to define statistical significance. Statistical analyses of tumors were conducted using SAS (SAS Institute Inc., Cary, NC, USA) version 9.4 and GraphPad Prism software.

## 4. Conclusions

As the leading diagnosed neoplasm in the US, NMSCs are medical, economic and social burdens, and are associated with increased complications such as inflammatory disorders and other cancers. Thus, it is crucial to find additional preventive measures to avoid damages caused by UVR, the leading cause of NMSC. As the chemoprevention field expands, more studies describe the beneficial effects of modifying dietary habits to prevent disease. We have demonstrated that consumption of dietary grape reduces UVB-mediated cutaneous damage and skin carcinogenesis in SKH-1 mice. We believe that antioxidant-rich dietary grape impairs early oxidative injury, leading to downstream anti-inflammatory effects and modulations in other pathways dysregulated in NMSC, resulting in a reduction in tumor growth and malignant conversion. As many differences are noted between males and females in the development of NMSC, this study complements our previous work demonstrating reduced tumorigenesis in female SKH-1 mice [24]. Indeed, further investigations, especially clinical trials, are required to validate these findings in human situations. If clinically validated, it is fairly achievable to modify dietary habits, with a grape-rich diet, for lowering the risk of skin cancers in human population.

## Supplementary Materials

The following is available online at https://www.mdpi.com/2072-6694/12/7/1751/s1, Figure S1: Full immunoblot images of bands shown in Figure 6.

## References

[1] Rogers H.W., Weinstock M.A., Feldman S.R., Coldiron B.M. (2015). Incidence estimate of nonmelanoma skin cancer (keratinocyte carcinomas) in the U.S. population, 2012. JAMA Dermatol..

[2] Apalla Z., Lallas A., Sotiriou E., Lazaridou E., Ioannides D. (2017). Epidemiological trends in skin cancer. Dermatol. Pract. Concept..

[3] Chren M.M., Torres J.S., Stuart S.E., Bertenthal D., Labrador R.J., Boscardin W.J. (2011). Recurrence after treatment of nonmelanoma skin cancer: A prospective cohort study. Arch. Dermatol..

[4] Armstrong B.K., Kricker A. (2001). The epidemiology of UV induced skin cancer. J. Photochem. Photobiol. B.

[5] Foote J.A., Harris R.B., Giuliano A.R., Roe D.J., Moon T.E., Cartmel B., Alberts D.S. (2001). Predictors for cutaneous basal- and squamous-cell carcinoma among actinically damaged adults. Int. J. Cancer.

[6] Guy G.P., Machlin S.R., Ekwueme D.U., Yabroff K.R. (2015). Prevalence and costs of skin cancer treatment in the U.S., 2002–2006 and 2007–2011. Am. J. Prev. Med..

[7] Heck D.E., Vetrano A.M., Mariano T.M., Laskin J.D. (2003). UVB light stimulates production of reactive oxygen species: Unexpected role for catalase. J. Biol. Chem..

[8] Afaq F., Adhami V.M., Ahmad N. (2003). Prevention of short-term ultraviolet B radiation-mediated damages by resveratrol in SKH-1 hairless mice. Toxicol. Appl. Pharmacol..

[9] Finkel T. (2011). Signal transduction by reactive oxygen species. J. Cell Biol..

[10] Ichihashi M., Ueda M., Budiyanto A., Bito T., Oka M., Fukunaga M., Tsuru K., Horikawa T. (2003). UV-induced skin damage. Toxicology.

[11] Taguchi M., Watanabe S., Yashima K., Murakami Y., Sekiya T., Ikedat S. (1994). Aberrations of the tumor suppressor p53 gene and p53 protein in solar keratosis in human skin. J. Investig. Dermatol..

[12] Melnikova V.O., Pacifico A., Chimenti S., Peris K., Ananthaswamy H.N. (2005). Fate of UVB-induced p53 mutations in SKH-hr1 mouse skin after discontinuation of irradiation: Relationship to skin cancer development. Oncogene.

[13] Wan Y.S., Wang Z.Q., Shao Y., Voorhees J.J., Fisher G.J. (2001). Ultraviolet irradiation activates PI 3-kinase/AKT survival pathway via EGF receptors in human skin in vivo. Int. J. Oncol..

[14] Chen W., Tang Q., Gonzales M.S., Bowden G.T. (2001). Role of p38 MAP kinases and ERK in mediating ultraviolet-B induced cyclooxygenase-2 gene expression in human keratinocytes. Oncogene.

[15] Kabuyama Y., Hamaya M., Homma Y. (1998). Wavelength specific activation of PI 3-kinase by UVB irradiation. FEBS Lett..

[16] Maru G.B., Gandhi K., Ramchandani A., Kumar G. (2014). The role of inflammation in skin cancer. Adv. Exp. Med. Biol..

[17] Taniguchi K., Karin M. (2018). NF-kappaB, inflammation, immunity and cancer: Coming of age. Nat. Rev. Immunol..

[18] Ulrich C., Jurgensen J.S., Degen A., Hackethal M., Ulrich M., Patel M.J., Eberle J., Terhorst D., Sterry W., Stockfleth E. (2009). Prevention of non-melanoma skin cancer in organ transplant patients by regular use of a sunscreen: A 24 months, prospective, case-control study. Br. J. Dermatol..

[19] Singh C.K., George J., Ahmad N. (2013). Resveratrol-based combinatorial strategies for cancer management. Ann. N. Y. Acad. Sci..

[20] Singh C.K., Ndiaye M.A., Ahmad N. (2015). Resveratrol and cancer: Challenges for clinical translation. Biochim. Biophys. Acta.

[21] Singh C.K., Liu X., Ahmad N. (2015). Resveratrol, in its natural combination in whole grape, for health promotion and disease management. Ann. N. Y. Acad. Sci..

[22] Singh C.K., Siddiqui I.A., El-Abd S., Mukhtar H., Ahmad N. (2016). Combination chemoprevention with grape antioxidants. Mol. Nutr. Food Res..

[23] Mintie C.A., Singh C.K., Ahmad N. (2020). Whole fruit phytochemicals combating skin damage and carcinogenesis. Transl. Oncol..

[24] Singh C.K., Mintie C.A., Ndiaye M.A., Chhabra G., Dakup P.P., Ye T., Yu M., Ahmad N. (2019). Chemoprotective effects of dietary grape powder on UVB radiation-mediated skin carcinogenesis in SKH-1 hairless mice. J. Investig. Dermatol..

[25] Reagan-Shaw S., Nihal M., Ahmad N. (2008). Dose translation from animal to human studies revisited. FASEB J..

[26] Benjamin C.L., Ullrich S.E., Kripke M.L., Ananthaswamy H.N. (2008). p53 tumor suppressor gene: A critical molecular target for UV induction and prevention of skin cancer. Photochem. Photobiol..

[27] D’Orazio J., Jarrett S., Amaro-Ortiz A., Scott T. (2013). UV radiation and the skin. Int. J. Mol. Sci..

[28] Siiskonen H., Smorodchenko A., Krause K., Maurer M. (2018). Ultraviolet radiation and skin mast cells: Effects, mechanisms and relevance for skin diseases. Exp. Dermatol..

[29] Varricchi G., Galdiero M.R., Marone G., Granata F., Borriello F., Marone G. (2017). Controversial role of mast cells in skin cancers. Exp. Dermatol..

[30] Che D.N., Xie G.H., Cho B.O., Shin J.Y., Kang H.J., Jang S.I. (2017). Protective effects of grape stem extract against UVB-induced damage in C57BL mice skin. J. Photochem. Photobiol. B.

[31] Thomas-Ahner J.M., Wulff B.C., Tober K.L., Kusewitt D.F., Riggenbach J.A., Oberyszyn T.M. (2007). Gender differences in UVB-induced skin carcinogenesis, inflammation, and DNA damage. Cancer Res..

[32] Benavides F., Oberyszyn T.M., VanBuskirk A.M., Reeve V.E., Kusewitt D.F. (2009). The hairless mouse in skin research. J. Dermatol. Sci..

[33] Missero C., Antonini D. (2017). p63 in squamous cell carcinoma of the skin: More than a stem cell/progenitor marker. J. Investig. Dermatol..

[34] Keyes W.M., Pecoraro M., Aranda V., Vernersson-Lindahl E., Li W., Vogel H., Guo X., Garcia E.L., Michurina T.V., Enikolopov G. (2011). ΔNp63α is an oncogene that targets chromatin remodeler Lsh to drive skin stem cell proliferation and tumorigenesis. Cell Stem Cell.

[35] Hanahan D., Weinberg R.A. (2011). Hallmarks of cancer: The next generation. Cell.

[36] Singh C.K., Chhabra G., Mintie C.A., Ahmad N., Pezzuto J.M., Vang O. (2020). Grape chemopreventive agents against angiogenesis and metastasis. Natural Products for Cancer Chemoprevention: Single Compounds and Combinations.

[37] Mintie C.A., Singh C.K., Ndiaye M.A., Barrett-Wilt G.A., Ahmad N. (2019). Identification of molecular targets of dietary grape-mediated chemoprevention of ultraviolet B skin carcinogenesis: A comparative quantitative proteomics analysis. J. Proteome Res..

[38] Sarchio S.N.E., Kok L.-F., O’Sullivan C., Halliday G.M., Byrne S.N. (2012). Dermal mast cells affect the development of sunlight-induced skin tumours. Exp. Dermatol..

[39] Falcone F.H., Haas H., Gibbs B.F. (2000). The human basophil: A new appreciation of its role in immune responses. Blood.

[40] Lupu M., Caruntu A., Caruntu C., Papagheorghe L.M.L., Ilie M.A., Voiculescu V., Boda D., Constantin C., Tanase C., Sifaki M. (2017). Neuroendocrine factors: The missing link in nonmelanoma skin cancer (Review). Oncol. Rep..

[41] Stone K.D., Prussin C., Metcalfe D.D. (2010). IgE, mast cells, basophils, and eosinophils. J. Allergy Clin. Immunol..

[42] Wiemels J.L., Wiencke J.K., Li Z., Ramos C., Nelson H.H., Karagas M.R. (2011). Risk of squamous cell carcinoma of the skin in relation to IgE: A nested case-control study. Cancer Epidemiol. Biomark. Prev..

[43] Han S.Y., Bae J.Y., Park S.H., Kim Y.H., Park J.H., Kang Y.H. (2013). Resveratrol inhibits IgE-mediated basophilic mast cell degranulation and passive cutaneous anaphylaxis in mice. J. Nutr..

[44] Leelahavanichkul K., Amornphimoltham P., Molinolo A.A., Basile J.R., Koontongkaew S., Gutkind J.S. (2014). A role for p38 MAPK in head and neck cancer cell growth and tumor-induced angiogenesis and lymphangiogenesis. Mol. Oncol..

[45] Voigt A.Y., Michaud M., Tsai K.Y., Oh J., Sundberg J.P. (2019). Differential hairless mouse strain-specific susceptibility to skin cancer and sunburn. J. Investig. Dermatol..

[46] Wang X., Seed B. (2003). A PCR primer bank for quantitative gene expression analysis. Nucleic Acids Res..

