# Supplementary material for: Protective Effects of Dietary Grape on UVB-Mediated Cutaneous Damages and Skin Tumorigenesis in SKH-1 Mice
Source: Cancers (Basel). 2020 Jul 1;12(7):1751. doi: 10.3390/cancers12071751 (PMC7409354; doi:10.3390/cancers12071751)
Supplement: Supplementary file 1 [file cancers-12-01751-s001.pdf]

Supplementary Material from:

Protective Effects of Dietary Grape on UVB-Mediated Cutaneous Damages and Skin Tumorigenesis in SKH-1 Mice

Charlotte A. Mintie [1], Anna K. Musarra [1], Chandra K. Singh [1], Mary A. Ndiaye [1], Ruth Sullivan [2], Jens C. Eickhoff [3] and Nihal Ahmad [1,4,*]

[1] Department of Dermatology, University of Wisconsin, 1300 University Avenue, Madison, WI 53706, USA; mintie@wisc.edu (C.A.M.); musarra@wisc.edu (A.K.M.); cksingh@wisc.edu (C.K.S.) mndiaye@dermatology.wisc.edu (M.A.N.)

[2] Department of Comparative Biosciences, University of Wisconsin, Madison, WI 53706, USA; rsulliv2@wisc.edu

[3] Department of Biostatistics & Medical Informatics, University of Wisconsin, Madison, WI 53705, USA; eickhoff@biostat.wisc.edu

[4] William S. Middleton VA Medical Center, Madison, WI 53705, USA

* Correspondence: nahmad@wisc.edu; Tel.: +1-608-263-2532

Received: 17 May 2020; Accepted: 28 June 2020; Published: date

Abstract: Non-melanoma skin cancers (NMSCs) are the most diagnosed cancers in the US and occur more frequently in males. We previously demonstrated chemoprotective effects of dietary grape powder (GP) against UVB-mediated skin tumorigenesis in female SKH-1 mice. To expand on this, here, we determined the effects of GP in a short-term UVB exposure protocol (0 or 5% GP, followed by UVB every other day for 2 weeks) in male and female SKH-1 mice, as well as explored any sex-related differences in UVB carcinogenesis via male SKH-1 mice (0, 3, or 5% GP; UVB twice weekly for 28 weeks). In the short-term study, we found that GP protects against early-stage epithelial hyperplasia and mast cell infiltration in both sexes. In the long term, GP markedly reduced tumor counts and malignant conversion, along with significant decreases in mast cell infiltration, serum IgE and Eotaxin. We also found inhibition of P38 phosphorylation and reduced PCNA, Ki67 and BCL2 levels, suggesting that the anti-inflammatory effects of GP inhibits P38, acting as an upstream regulator to inhibit proliferation and reduce tumor cell survival. Together, GP appears to protect against UVB-mediated skin damage and carcinogenesis in SKH-1 mice and should be explored further as a supplement for NMSC prevention.

Keywords: non-melanoma skin cancer; grape; SKH-1; UVB; chemoprevention

Western Blot Images from Figure 6

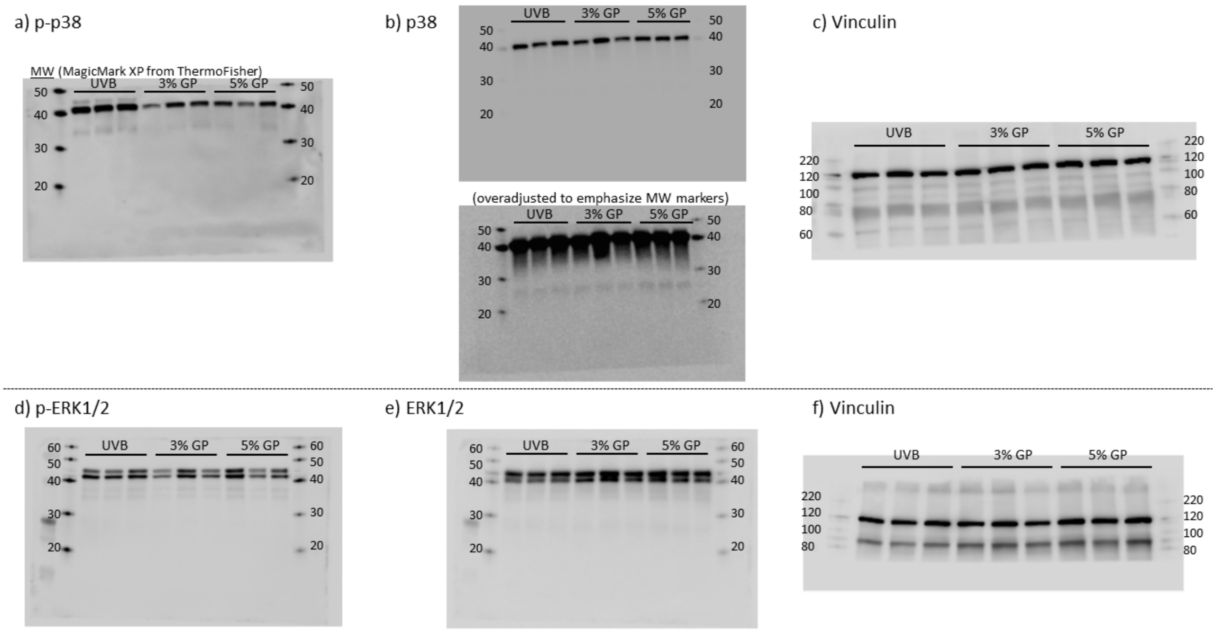

Figure S1. Full immunoblot images of bands shown in Figure 6. Full images of the phosphorylated p38 (p-p38; a), total p38 (b), phosphorylated ERK1/2 (p-ERK1/2; d), total ERK1/2 (E) immmunoblots along with vinculin images (c,f) used as loading controls. Numbers along the sides indicate the molecular weight standard sizes (in kDa).
